# Are IQ and educational outcomes in teenagers related to their cannabis use? A prospective cohort study

**DOI:** 10.1177/0269881115622241

**Published:** 2016-02

**Authors:** C Mokrysz, R Landy, SH Gage, MR Munafò, JP Roiser, HV Curran

**Affiliations:** 1 Clinical Psychopharmacology Unit, University College London, London, UK; 2 Centre for Cancer Prevention, Wolfson Institute of Preventive Medicine, Queen Mary University of London, London, UK; 3 MRC Integrative Epidemiology Unit, University of Bristol, Bristol, UK; 4 Institute of Cognitive Neuroscience, University College London, London, UK

**Keywords:** Cannabis, IQ, education, ALSPAC, cigarettes

## Abstract

There is much debate about the impact of adolescent cannabis use on intellectual and educational outcomes. We investigated associations between adolescent cannabis use and IQ and educational attainment in a sample of 2235 teenagers from the Avon Longitudinal Study of Parents and Children. By the age of 15, 24% reported having tried cannabis at least once. A series of nested linear regressions was employed, adjusted hierarchically by pre-exposure ability and potential confounds (e.g. cigarette and alcohol use, childhood mental-health symptoms and behavioural problems), to test the relationships between cumulative cannabis use and IQ at the age of 15 and educational performance at the age of 16. After full adjustment, those who had used cannabis ⩾50 times did not differ from never-users on either IQ or educational performance. Adjusting for group differences in cigarette smoking dramatically attenuated the associations between cannabis use and both outcomes, and further analyses demonstrated robust associations between cigarette use and educational outcomes, even with cannabis users excluded. These findings suggest that adolescent cannabis use is not associated with IQ or educational performance once adjustment is made for potential confounds, in particular adolescent cigarette use. Modest cannabis use in teenagers may have less cognitive impact than epidemiological surveys of older cohorts have previously suggested.

## Introduction

Cannabis use typically starts in adolescence, with heaviest use generally reported during the teenage years (Chen and Kandel, 1995). An estimated 17% of 15–16 year olds in Europe and 34% in the United States have taken cannabis at least once (Hibell et al., 2012; Miech et al., 2015). In the previous month, cannabis was used by 7% (Europe) and 17% (US) of this age group, which compares to 57% (Europe) and 24% (US) using alcohol and 28% (Europe) and 7% (US) using cigarettes (Hibell et al., 2012; Miech et al., 2015). With current global debates around legalisation and medical uses of cannabis, research on whether the drug has detrimental cognitive effects, particularly for adolescents, is critically important for policy decisions.

Acutely, cannabis induces robust and dose-dependent episodic memory impairments (Curran et al., 2002; Ranganathan and D’Souza, 2006), with more mixed reports of impaired working memory, attention, psychomotor control, inhibition and abstract reasoning (Crane et al., 2013; Crean et al., 2011; Gonzalez, 2007). More debated is whether there are persistent cognitive effects of using cannabis. Although long-lasting neuropsychological impairments have been reported (Crane et al., 2013; Crean et al., 2011; Gonzalez, 2007; Grant et al., 2003; Pope et al., 2003; Ranganathan and D’Souza, 2006), many studies have been cross-sectional and cannot exclude the possibility of pre-existing group differences in cognitive ability.

To date, two longitudinal investigations have specifically assessed the link between cannabis use and IQ. One small-scale study of young adults (*N*=113, including 59 never-users of cannabis; Fried et al., 2005) found evidence of lower IQ in current but not former cannabis users. Recently, a prospective analysis of the Dunedin Longitudinal Study cohort (born 1972–1973; *N*=874, including 242 never-users of cannabis) found evidence of IQ decline in 38 year olds who became cannabis dependent after starting weekly cannabis use in adolescence, but not in those who became regular users later in adulthood (Meier et al., 2012). The decline in IQ remained apparent even for those who were no longer regular cannabis users at the time of adult IQ assessment. These findings highlight the growing concern that *adolescent* cannabis use may be particularly detrimental to cognitive development. However, determining causality is challenging, since we cannot assume that cannabis users and non-users would have developed along similar trajectories if cannabis use had not occurred (Rogeberg, 2013). Adolescents who use cannabis regularly also tend to have higher rates of social adversity (von Sydow et al., 2002), early-onset behavioural problems (Heron et al., 2013) and other adolescent substance use (Hibell et al., 2012), all of which may confound the relationships between cannabis use and poorer intellectual and educational outcomes.

Relatedly, there is a wide evidence base linking adolescent cannabis use to early school leaving and poorer educational performance (Fergusson et al., 2003; Lynskey and Hall, 2000; Lynskey et al., 2003; McCaffrey et al., 2010; Silins et al., 2014; Stiby et al., 2014). Typically, these associations are robust to adjustment for potential confounds, and one explanation for these findings is that cannabis use negatively impacts on cognitive ability and therefore academic performance. However, the possibility of group differences in pre-exposure school performance is often not addressed, and adjustment for other related adolescent behaviours, such as use of other substances, has been limited. As such, the existence of unmeasured confounds is often posited as an explanation of negative associations with cannabis (Fergusson et al., 2003; Lynskey and Hall, 2000; McCaffrey et al., 2010). Indeed, this is supported by recent genetic studies that found no difference in early school leaving between both monozygotic and dizygotic twin pairs who were discordant for cannabis use (Grant et al., 2012; Verweij et al., 2013).

Here, we examined the associations between adolescent cannabis use and both IQ and educational attainment within a large adolescent cohort sample. Assessing intellectual and educational outcomes in the same longitudinal cohort, with similar confounder adjustment, enables better integration of findings across both domains. We considered several factors commonly associated with teenage cannabis use that may account for previously reported associations with IQ and educational performance. In particular, we addressed the role of other drug use, using detailed measures of cigarette, alcohol and other recreational drug use. We hypothesised that cannabis use would be associated with both IQ and educational performance. However, these associations may be attenuated by adjusting for confounders.

## Methods

### Sample

Participants were members of the Avon Longitudinal Study of Parents and Children (ALSPAC) cohort, a prospective study in Bristol (UK) following women and their children since pregnancy. ALSPAC recruited pregnant mothers from the former Avon Health Authority, England, with an expected delivery date from April 1991 to December 1992. The core cohort comprised 14,541 pregnancies, with 13,988 babies alive at one year (Boyd et al., 2013). Following later recruitment of eligible cases that were not initially recruited, the cohort now comprises 15,458 foetuses, with 14,701 babies alive at one year. IQ scores at both 8 and 15 years of age were available for 4621 participants. Of these, and after exclusions (*n*=41: mother reported child head injury resulting in unconsciousness, *n*=38; child indicated use of imaginary drug ‘spanglers’ (a fictional drug included in the questionnaire to test veracity of participants’ responses) since 15th birthday, *n*=3), 2235 individuals had complete data for all key variables and confounders, and so were included in the main analyses (complete-case sample; see Figure S1 for participant flow diagram). The study website contains details of all available data through a fully searchable data dictionary (http://www.bris.ac.uk/alspac/researchers/data-access/data-dictionary). Ethical approval was obtained from ALSPAC Law and Ethics Committee.

### Measures

#### Cannabis use

Participants provided cumulative lifetime cannabis use data at the age of 15, via a self-report questionnaire administered during attendance at clinic sessions. Initial responses were categorical, with six levels: ‘never’, ‘less than 5 times’, ‘5–19 times’, ‘20–49 times’, ‘50–99 times’ and ‘100 times or more’. For the present study, sample size considerations resulted in the two highest levels being combined into one response level of ‘50 times or more’, creating a five-level categorical variable of cumulative cannabis use.

#### IQ

Participants were administered the Wechsler Intelligence Scale for Children 3rd Edition (WISC-III; Wechsler, 1991) at an individual clinic session at the age of 8. Alternate items of the WISC-III were administered for all sections, apart from the coding subtest for which all items were included. IQ measurements were calculated for each individual, adjusting for age. At the age of 15, participants were administered the Vocabulary and Matrix Reasoning subsections of the Wechsler Abbreviated Scale of Intelligence (WASI; Wechsler, 1999). IQ was again calculated for each individual, adjusting for age. To ease interpretation, IQ scores were rescaled around the complete-case sample included in the present analysis, to a mean of 100 and standard deviation of 15.

#### Educational performance

In England, children attending state-maintained schools are educated in line with the National Curriculum, which defines what subjects must be taught and the standards children should reach at each stage. The Curriculum is split into a series of ‘Key Stages’, which are assessed by compulsory teacher assessments or national tests at the end of each stage (for further information, see www.gov.uk/national-curriculum/overview). Data linkage between ALSPAC and the National Pupil Database (a central repository for pupil-level educational data in England) provided educational assessment data for participants who attended state-funded schools at Key Stages 2 (age 11) and 4 (age 16). Data linkage was performed by a third-party company and checked by the ALSPAC team (for further information, see www.adls.ac.uk/department-for-education/dcsf-npd/?detail). Raw scores at the age of 11, when children sit Key Stage 2 tests for Maths, English and Science, were converted to percentages and averaged across the three subjects. Educational performance at the age of 16, when pupils complete Key Stage 4 national testing, was quantified using a standard capped scoring method (see http://nationalpupildatabase.wikispaces.com/KS4) in which grades achieved at General Certificate of Secondary Education (GCSE) or equivalent for their best eight subjects are converted to a numerical score (e.g. A*=58 points … G=16 points) and summed. Capped scores, out of a maximum possible score of 464, were then converted to a percentage.

### Potential confounds

Potential confounds were chosen in accordance with theoretical considerations and previous literature to reflect variables associated with adolescent cannabis use and intellectual and educational outcomes: 1. *maternal and early-life factors* (Fergusson et al., 1990; Fergusson and Horwood, 1997; Heron et al., 2013; von Sydow et al., 2002): maternal education [none/Certificate of Secondary Education, O-levels, A-levels, degree]; child sex; maternal depressive symptoms during pregnancy and up to eight months postnatal [mother completed depression items of the Crown–Crisp experiential index (CCEI); Crisp et al., 1978]; maternal substance use during the first three months of pregnancy [alcohol use: none, less than weekly, at least weekly; cigarette use: no, yes; cannabis use: no, yes]; 2. *childhood behavioural factors* (Heron et al., 2013; Lynskey and Fergusson, 1995; Shedler and Block, 1990): hyperactivity and conduct problems at age 11 [mother completed Strengths and Difficulties Questionnaire; Goodman, 1997]; mother suspected truancy at age 14 [no, yes]; 3. *childhood mental health* (Degenhardt et al., 2003; Patton et al., 2002): depressive symptoms at age 12 [child completed depression items of the Short Mood and Feelings Questionnaire; Angold et al., 1995]; psychotic-like symptoms at age 12 [child completed PLIKSi semi-structured interview; Zammit et al., 2013]; 4. *other adolescent drug use* (Chamberlain et al., 2012; Fergusson and Horwood, 2000; Fergusson et al., 2006; Patton et al., 2005; Rob et al., 1990; Stiby et al., 2014): cumulative cigarette use self-reported at age 15 [never, 1–4, 5–20, 21–60, 61–100, >100 times]; cumulative alcohol use self-reported at age 15 [never, 1–5, 6–19, 20–39, 40–99, ⩾100 times]; and other recreational drug use since 15th birthday, including ketamine, LSD, cocaine, ecstasy, amphetamine and inhalants, self-reported at age 15 [none, used one other drug, used more than one other drug].

### Statistical analyses

Analyses were conducted using Stata/SE v13.1 (StataCorp LP, College Station, TX). A series of nested linear least-squares regression analyses was employed and adjusted by potential confounders to test the relationships between cumulative cannabis use and (a) IQ at the age of 15 and (b) educational performance at the age of 16.

Unadjusted estimates of the relationship between cumulative cannabis use (dummy-coded from 1 to 5, representing the categories explained above) and IQ age 15 (Model IQ1) were compared to adjusted estimates derived from a series of nested models that additionally included: first, pre-exposure IQ at the age of 8 (Model IQ2); then, in addition to IQ2, maternal, early-life and childhood behavioural factors (Model IQ3); then, in addition to IQ3, adolescent mental-health factors (Model IQ4); then, in addition to IQ4, cigarette use (Model IQ5a), alcohol use (Model IQ5b), or other drug use (Model IQ5c); finally, a fully adjusted model (Model IQ6) which included all potential confounds.

Unadjusted estimates of the relationship between cumulative cannabis use (again dummy-coded from 1 to 5, representing the categories explained above) and educational performance at the age of 16 (Model Ed1) were compared to adjusted estimates derived from a series of nested models that initially included educational performance at age 11 (Model Ed2). Models Ed3–6 were then adjusted as for Models IQ3–6.

### Multiple imputation analyses

For clarity, we have focused primarily on the results of the complete-case analyses. However, to supplement these findings, we repeated planned analyses after implementing multiple imputation with chained equations to account for missing data (20 imputations, using the *ice* command in Stata). Multiple imputation was carried out for all participants alive at one year, resulting in a sample size of 14,552 after multiple imputation and exclusions. This method assumes data are missing at random (i.e. that the probability of a data point being missing depends only on observed data). Previously described guidelines were followed when selecting variables for the imputation model from the many variables collected by ALSPAC (Van Buuren et al., 1999). Due to the large number of variables that met these criteria, different sets of variables were selected for the imputation of groups of variables. Data were imputed for all outcome, key predictor and confounder variables. Full details of variables included in the imputation model are available from the authors on request.

### Post hoc analyses

Post hoc linear least-squares regression analyses were then employed and adjusted by potential confounders to test the relationships between cumulative cigarette use and (a) IQ at the age of 15 and (b) educational performance at the age of 16, after exclusion of cannabis users. Unadjusted estimates of the relationship between cumulative cigarette use (binary outcome due to smaller sample: never used/ever used cigarettes) and (a) IQ age 15 (Model CigIQ1) were compared to fully adjusted estimates (Model CigIQ2), and (b) educational performance at the age of 16 (Model CigEd1) were compared to fully adjusted estimates (Model CigEd2).

## Results

Of the complete-case data set (*n*=2235), 23.5% (*n*=526) reported having tried cannabis at least once, and 3.3% (*n*=74) reported cumulative usage of at least 50 times. Table 1 shows the demographics of the sample according to reported cumulative cannabis use at an average age of 15.4 years. Unadjusted analyses demonstrate that cannabis use was associated with maternal cigarette and cannabis use during pregnancy, truancy from school, childhood hyperactivity, conduct problems and depressive symptoms, and adolescent cigarette, alcohol and other drug use.

**Table 1.** Demographic and baseline variables for each cannabis use group.

	**Never% (*n*)**	**<5% (*n*)**	**5–19% (*n*)**	**20–49% (*n*)**	**⩾50% (*n*)**	***p*-Value**
Sample (complete-cases)	76.5 (1709)	11.1 (248)	6.0 (133)	3.2 (71)	3.3 (74)	
Female	53.5 (914)	59.7 (148)	52.6 (70)	46.5 (33)	39.2 (29)	0.060
Mother had no higher education	80.2 (1371)	85.1 (211)	77.4 (103)	70.4 (50)	77.0 (57)	0.171
Cigarette use during first three months of pregnancy	10.5 (179)	18.6 (46)	22.6 (30)	23.9 (17)	33.8 (25)	⩽0.001
Weekly alcohol use during first three months of pregnancy	13.5 (231)	14.9 (37)	20.3 (27)	16.9 (12)	16.2 (12)	0.074
Cannabis use during first three months of pregnancy	0.9 (16)	2.0 (5)	5.3 (7)	4.2 (3)	8.1 (6)	⩽0.001
Truancy from school, age 14	0.7 (12)	2.4 (6)	3.8 (5)	9.9 (7)	6.8 (5)	⩽0.001
Lifetime cigarette use >20 times, age 15	4.5 (77)	34.3 (85)	52.6 (70)	71.8 (51)	83.8 (62)	⩽0.001
Lifetime alcohol use >20 times, age 15	26.4 (452)	63.7 (158)	77.4 (103)	71.8 (66)	97.3 (72)	⩽0.001
Other illicit drug use, since 15th birthday	5.7 (97)	28.6 (71)	43.6 (58)	54.9 (39)	67.6 (50)	⩽0.001
	**Mean (SE)**	**Mean (SE)**	**Mean (SE)**	**Mean (SE)**	**Mean (SE)**	***p*-Value**
IQ score age 8	99.7 (0.4)	100.3 (1.0)	101.6 (1.2)	101.7 (1.9)	102.0 (1.7)	0.335
Educational performance, age 10/11	73.2 (0.3)	73.4 (0.8)	73.3 (1.0)	70.0 (1.8)	72.3 (1.5)	0.202
Maternal depressive symptoms	3.6 (0.1)	3.5 (0.1)	4.0 (0.2)	4.1 (0.3)	3.9 (0.2)	0.050
Hyperactivity, age 11	2.4 (0.1)	2.53 (0.1)	2.5 (0.2)	3.5 (0.3)	3.4 (0.3)	⩽0.001
Conduct problems, age 11	1.0 (0.0)	1.2 (0.1)	1.2 (0.1)	1.7 (0.2)	1.4 (0.2)	⩽0.001
Childhood depressive symptoms, age 12	4.3 (0.0)	4.9 (0.3)	5.0 (0.4)	5.1 (0.5)	6.6 (0.7)	⩽0.001
Childhood psychotic-like symptoms, age 12	0.3 (0.0)	0.2 (0.1)	0.3 (0.1)	0.4 (0.1)	0.3 (0.1)	0.549

*Note: p*-Values reflect omnibus test of cannabis use group differences.

Table 2 shows patterns of cannabis use according to cumulative use groups. Within those who had tried cannabis at least once, greater exposure was associated with a younger age of first cannabis use (*p*<0.001) and a longer time since first usage (*p*<0.001). Those who had ⩾50 cannabis exposures had first used at a mean age of 13.1 years, and for a mean duration of 2.3 years. Of those with ⩾50 exposures, 98.7% had used in the past year, 60.8% were currently using at least weekly and 47.3% had used in the three days prior to the IQ test. The majority (91.0%) usually mixed tobacco with their cannabis.

**Table 2.** Cannabis use patterns for each cannabis use group.

	<5	5–19	20–49	⩾50	*p*-Value
Sample (complete-cases; cannabis users)	47.1 (248)	25.3 (133)	13.5 (71)	14.1 (74)	
Age first tried cannabis, years, mean (SE)	14.3 (0.1)	14.0 (0.1)	13.4 (0.1)	13.1 (0.1)	⩽0.001
Time since first cannabis use at time of IQ test, years, mean (SE)	1.1 (0.1)	1.4 (0.1)	2.0 (0.1)	2.3 (0.1)	⩽0.001
Currently uses cannabis at least weekly	0.0 (0)	3.8 (5)	23.9 (17)	60.8 (45)	⩽0.001
Has used cannabis in past year	62.0 (153)	94.0 (125)	90.1 (64)	98.7 (73)	⩽0.001
Used cannabis in the past three days at time of IQ test	2.4 (6)	6.8 (9)	23.9 (17)	47.3 (35)	⩽0.001
Usually smokes cannabis mixed with tobacco^a^	90.7 (127)	87.7 (107)	93.0 (66)	94.5 (69)	0.272
Typically smokes 1/16th ounce (‘£10 bag’) in less than one day^a^	14.3 (11)	18.1 (19)	30.2 (19)	38.9 (28)	⩽0.001

Note: Values are % (*n*) unless otherwise noted. *p*-Values reflect omnibus test of cannabis use group differences.

a Excluding those who answered ‘don’t know’.

### IQ

**Figure 1.**
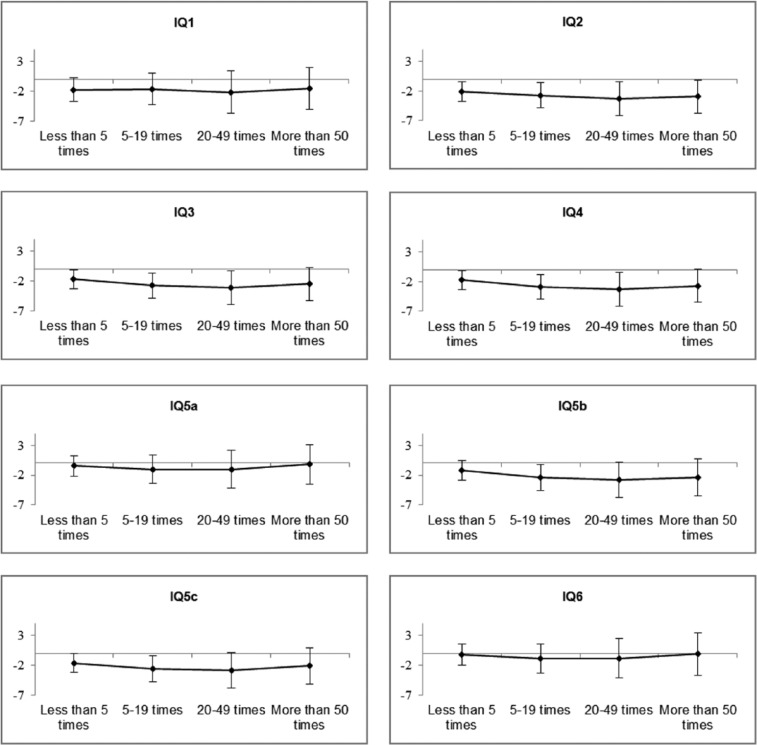
Linear regression nested models for complete-cases data set displaying difference in IQ scores at the age of 15, estimated between each cannabis use group compared to never-users. Error bars represent 95% confidence intervals.

Unadjusted IQ data for the cannabis use groups can be found in Table 3. Model estimates are displayed in Figure 1 and Table S1. In the unadjusted analyses, no difference in IQ between the cannabis use groups was apparent (*p*=0.237). However, after adjusting for IQ measured at the age of 8, cumulative cannabis use was negatively associated with IQ measured at the age of 15 (Model IQ2; *p*<0.001). Those who had used cannabis ⩾50 times were estimated to have an IQ at the age of 15 that was 2.9 points lower than never-users in this model. Adjustment by maternal, early-life and childhood behavioural factors (Model IQ3) and by mental-health factors (Model IQ4) had little effect on point estimates. Adjustment for cigarette (Model IQ5a), alcohol (Model IQ5b) or other substance use (Model IQ5c) attenuated the association between cannabis use and IQ at the age of 15, with cigarette use having the most marked influence. Model IQ6 fully attenuated the association between cannabis use and IQ at the age of 15 (*p*=0.959), with cumulative use of ⩾50 times now predicting an adjusted IQ score of 0.1 (*p*=0.941) lower relative to never-users.

**Table 3.** Mean and 95% confidence intervals of IQ at the age of 15 and educational performance (% GCSE points) at the age of 16 for each of the cannabis use groups.

Cumulative cannabis use	% (*N*)	WASI IQ (age 15)			Educational performance % (age 16)		
		Mean	95% CIs		Mean	95% CIs	
			Lower	Upper		Lower	Upper
Never	76.5 (1709)	100.4	99.7	101.1	80.8	80.2	81.4
<5	11.1 (248)	98.6	96.8	100.5	77.8	76.2	79.4
5–19	6.0 (133)	98.8	96.2	101.4	76.5	73.9	79.1
20–49	3.2 (71)	98.3	94.6	101.9	72.8	68.8	76.8
⩾50	3.3 (74)	98.9	95.6	102.2	69.2	65.0	73.3
Overall	100.0 (2235)	100.0	99.4	100.6	79.6	79.0	80.1

CI: confidence intervals; WASI: Wechsler Abbreviated Scale of Intelligence.

### Educational performance

**Figure 2.**
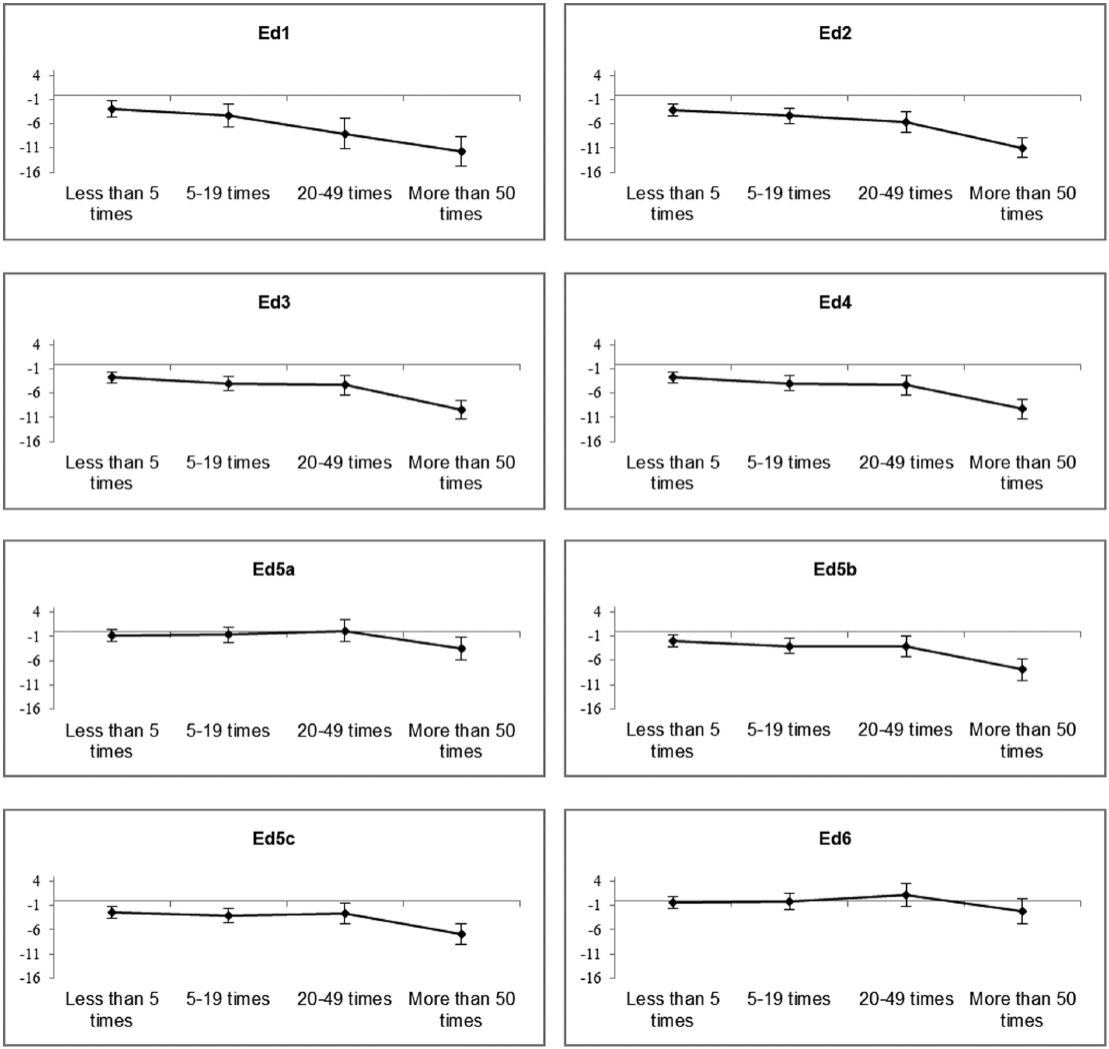
Linear regression nested models for complete-cases data set displaying difference in educational performance at the age of 16, estimated between each cannabis use group compared to never-users. Error bars represent 95% confidence intervals.

Unadjusted educational performance data for the cannabis use groups are shown in Table 3. Model estimates are displayed in Figure 2 and Table S2. Increasing cumulative cannabis use correlated with poorer educational performance at the age of 16 (*p*<0.001). Cannabis use of ⩾50 times predicted an average score of 11.6 percentage points lower than never-users (*p*<0.001). After adjusting for educational performance at the age of 11, cannabis use remained associated with educational performance at the age of 16 (Model Ed2; *p* < 0.001); those who had used cannabis ⩾50 times were estimated to have scored 11.0 percentage points lower than never-users (*p* < 0.001). Adjustment by maternal, early-life and childhood behavioural factors (Model Ed3) and mental-health factors (Model Ed4) had little effect on point estimates. Adjustment by alcohol (Model Ed5a), cigarette (Model Ed5b) or other substance use (Model Ed5c) attenuated the association between cannabis use and educational performance at the age of 16, with cigarette use again having the most marked influence. Model Ed6 fully attenuated the association between cannabis use and educational performance at the age of 16 (*p*=0.184), with cumulative use ⩾50 times now predicting an adjusted score of 2.2 (*p*=0.083) percentage points lower than never-users.

### Cigarettes

In the above analyses, cumulative cigarette use was the key attenuator of the association between cumulative cannabis use and both IQ (see Models IQ5a and IQ6) and educational performance (see Models Ed5a and Ed6). Further, cumulative cigarette use remained negatively associated with both outcomes in the fully adjusted models. Those who had used cigarettes >100 times were estimated to have an age 15 adjusted IQ 3.2 points lower (*p*=0.018) and an adjusted educational score 7.4 percentage points lower (*p*<0.001) than never-users of cigarettes (see Tables S3 and S4).

To explore these relationships further, we investigated associations with cigarette use in those who had never used cannabis (Table 4a). Of the complete-case sample, 76.4% (*n*=1709) had never tried cannabis, of which 13.9% (*n*=237) reported having tried cigarettes at least once. Analyses were repeated on this restricted sample with ever-use of cigarettes as the primary predictor (Table 4b). With adjustment for only pre-exposure IQ or educational performance, respectively, ever-use of cigarettes was associated with an age 15 IQ 6.2 points lower (*p*<0.001; Model CigIQ1), and educational performance 7.8 percentage points lower (*p*<0.001; Model CigEd1), relative to never-users of cigarettes. After full adjustment, these relationships were somewhat attenuated, with ever-use of cigarettes now predicting an age 15 adjusted IQ 1.5 points lower (*p*=0.083; Model CigIQ2), and educational performance 2.9 percentage points lower (*p*<0.001; Model CigEd2), relative to never-users of cigarettes.

**Table 4.** (a) Mean and 95% CIs of IQ at the age of 15 and educational performance at the age of 16 (% GCSE points) for ever-users of cigarettes (*n*=237) compared to never-users of cigarettes (*n*=1472).

Ever-use of cigarettes	% (*N*)	WASI IQ (age 15)			Educational performance % (age 16)		
		Mean	95% CIs		Mean	95% CIs	
			Lower	Upper		Lower	Upper
Non-users	86.1 (1472)	101.3	100.5	102.0	81.9	81.2	82.5
Tried cigarettes at least once	13.9 (237)	95.1	93.4	96.8	74.1	72.5	75.7

Note: Cannabis users were excluded from this analysis.

**Table:** (b) Linear regression nested models displaying difference in IQ at the age of 15 and educational performance at the age of 16 estimated for ever-users of cigarettes (*n*=237) compared to never-users of cigarettes (*n*=1472).

	IQ		Education	
	Model CigIQ1	Model CigIQ2	Model CigEd1	Model CigEd2
Tried cigarettes at least once	−6.2 (1.0)**	−1.5 (0.9)*	−7.8 (0.8)**	−2.9 (0.6)**

Note: Cannabis users were excluded from this analysis.

* Two-tailed *t*-tests, compared to never-users (*p*=0.083).

** Two-tailed *t*-tests, compared to never-users (*p*<0.01).

### Multiple imputation analyses

Key variables were compared for participants who were included in the complete-case analysis and the available data for the participants who had missing data but were alive at one year (Table S5). Drop-out was related to a range of variables, including pre-cannabis exposure IQ score at the age of 8. To investigate whether drop-out may have influenced the results, we conducted multiple imputation to supplement the main analyses, resulting in a large imputed data set (*n*=14,552).

Point estimates and the patterns of attenuation observed after adjusting hierarchically for potential confounds were similar for the complete and imputed case analyses. However, while for the unadjusted complete-case IQ analysis (Model IQ1) there was no difference in IQ between cannabis use groups, for the unadjusted imputed analysis lower scores were associated with greater cannabis use (Table S6, Model IQ1i). Additionally, for the complete-case education analyses, adjustment for cigarette use (Models Ed5a) did not fully attenuate the association between cannabis use and educational performance. However, in the imputed analyses, this association was fully attenuated (Table S7, Models Ed5ai).

## Discussion

In line with previous work, we found that cannabis users had lower teenage IQ scores and poorer educational performance than teenagers who had never used cannabis. At the same time, cannabis users also had higher rates of childhood behavioural problems, childhood depressive symptoms, other substance use (including use of cigarettes and alcohol) and maternal use of cannabis during pregnancy. After adjustment to account for these group differences, cannabis use by the age of 15 did not predict either lower teenage IQ scores or poorer educational performance. These findings therefore suggest that cannabis use at the modest levels used by this sample of teenagers is not by itself causally related to cognitive impairment. Instead, our findings imply that previously reported associations between adolescent cannabis use and poorer intellectual and educational outcomes may be confounded to a significant degree by related factors.

While we found no evidence of a robust link between adolescent cannabis use and IQ, previous work has indeed shown that persistent cannabis dependence starting with regular cannabis use in adolescence is associated with IQ decline by middle age (Meier et al., 2012). Together, these findings suggest that while persistent cannabis dependence may be linked to declining IQ across a person’s lifetime, teenage cannabis use alone does not appear to predict worse IQ outcomes in adolescents. The present findings also highlight the importance of considering other adolescent substance use alongside cannabis, in particular cigarette use, when assessing links between cannabis and intellectual outcomes, and this confound may contribute to previously reported associations between cannabis dependence and IQ decline. However, the young age at which our outcomes were measured, and the relatively modest levels of cannabis use in the present sample, do not allow us to rule out the possibility of future difficulties, perhaps following further cannabis exposure. Assessing outcomes at this young age, before the end of compulsory education, does however have the benefit of reducing the potentially confounding influence of selection into or out of cognitively demanding environments throughout a person’s life on IQ performance (Rogeberg, 2013).

Attenuation of the association between cannabis use and educational performance contrasts with previous work demonstrating a robust relationship even after adjustment for confounders (Lynskey and Hall, 2000; Lynskey et al., 2003; Silins et al., 2014; Stiby et al., 2014). Notably, however, previous work reporting associations between cannabis use and poorer educational outcomes has not consistently addressed the possibility of group differences in pre-exposure educational performance or in rates of other substance use, which may explain differences between our findings and previous work. Indeed, our findings are in accordance with recent genetic studies that found no difference in early school leaving between both monozygotic and dizygotic twin pairs who were discordant for cannabis use (Grant et al., 2012; Verweij et al., 2013).

Compared with those in our sample who had never tried cannabis, teenagers who had used cannabis at least 50 times were 17 times more likely (84% vs. 5%) to have smoked cigarettes more than 20 times in their lifetime. Accounting for group differences in cigarette smoking dramatically attenuated the associations between cannabis use and both IQ and educational performance. Further, even after excluding those who had never tried cannabis, cigarette users were found to have lower educational performance (adjusted performance 2.9 percentage points lower, approximately equivalent to dropping two grades on one subject taken at GCSE) relative to those who had never tried cigarettes. A relationship between cigarette use and poorer cognitive (Chamberlain et al., 2012; Hooper et al., 2014; Weiser et al., 2010; Whalley et al., 2005) and educational (McCaffrey et al., 2010; Silins et al., 2014; Stiby et al., 2014) outcomes has been noted previously, and may have a number of explanations. Cigarette use may have a negative impact on cognitive ability. However, this is not supported by the experimental psychopharmacology literature, which robustly shows that acute nicotine administration results in transient cognitive enhancement (Heishman et al., 2010). Alternatively, reverse causality may contribute to this relationship, for example performing poorly at school may lead to increased engagement in risky behaviours such as cigarette smoking. Further, residual confounding may contribute to this link: cigarette smoking may be a marker of unmeasured factors, for example social adversity during adolescence, that influence both IQ and educational attainment.

Overwhelmingly, the most common method of cannabis administration was smoking it combined with tobacco (as is typical in the UK), potentially making it difficult to separate the independent contributions of cannabis and tobacco use on the outcomes. However, as noted above, lower educational performance remained apparent for cigarette smokers who had never used cannabis, even following adjustment for potential confounders. This suggests that it may be cigarette use, rather than tobacco consumption per se, that predicts poorer educational outcomes, potentially lending support to a non-pharmacological mechanism to explain links between cigarette use and poorer outcomes. Cigarette use has recently been highlighted as an important factor when exploring links between cannabis use and various outcomes, including psychotic-like experiences (Gage et al., 2014), educational outcomes (Stiby et al., 2014) and cannabis dependence (Hindocha et al., 2015). The relationship between cannabis and tobacco and/or cigarette use is complex, and there is a need to delineate the contribution of these substances when used alone and in combination (Schuster et al., 2015). This would be helped by improved measures of tobacco and cannabis consumption, and by comparing findings from cohorts with differing degrees of combined cannabis/tobacco administration.

A number of measurement limitations of the present study should be noted. Firstly, classification of cannabis users into groups based on self-reported cumulative uses does not provide information on actual dose (of delta-9-tetrahydrocannabinol; THC), which varies according to cannabis weight and particular strain, as well as how the user titrates the dose (Freeman et al., 2014; Temple et al., 2011; van der Pol et al., 2014). This is a limitation of all cohort-based research to date, since objective biological markers (e.g. of cannabinoids in hair) are not typically collected. It is noteworthy that the ALSPAC cohort, born in the early nineties, may have been exposed to higher THC potency varieties than earlier cohorts, which may be expected to have made cognitive impairment more rather than less likely. Secondly, an abbreviated WASI was used for IQ assessment at the age of 15, which provides a less reliable estimate of IQ than full-scale tests (Axelrod, 2002). However, as all the participants completed the same assessments, our comparisons remain valid.

Further, nearly half of those who had used cannabis at least 50 times reported having used it in the three days prior to their age 15 IQ assessments. Experimental research with humans has suggested that cognitive impairments seen up to six hours after acute oral doses of THC do not persist 24 or 48 hours later (Curran et al., 2002). However, a recent meta-analysis demonstrates that residual cognitive effects of chronic cannabis exposure may last approximately one month following abstinence from the drug (Schreiner and Dunn, 2012). Sample-size considerations meant that we could not assess the impact of excluding recent cannabis smokers from analyses, but future work, perhaps with the ALSPAC sample at an older age, should address the possibility of residual effects directly. Nevertheless, despite high levels of recent cannabis usage, we found no robust association between cannabis use and poorer IQ performance.

In summary, the notion that cannabis use itself is causally related to lower IQ and poorer educational performance was not supported in this large teenage sample. However, this study indeed has limitations, in particular the young age of outcome assessment. While we have demonstrated that confounding may be an explanation for links between cannabis use and poorer outcomes, large prospective cohorts tracking young people prior to, during and after stopping cannabis use, using more objective measures of drug use (e.g. the new NIH-funded ‘ABCD study’ in the United States; National Institute on Drug Abuse, 2015) are required before we can make strong conclusions. Cigarette smoking in particular has once again (Hooper et al., 2014; McCaffrey et al., 2010; Silins et al., 2014; Stiby et al., 2014) been highlighted as an important factor in adolescent outcomes, as well as a robust independent predictor of educational performance, and the reasons for this need to be elucidated. With ongoing debates about cannabis and tobacco legislation around the world, and the impact this could have on drug availability to teenagers, it is important that we can provide accurate information about drug harms regardless of whether the drug in question is legal or not.

## Supplementary Material

Supplementary material
