# Supplementary material for: Are IQ and educational outcomes in teenagers related to their cannabis use? A prospective cohort study
Source: J Psychopharmacol. 2016 Feb;30(2):159–68. doi: 10.1177/0269881115622241 (PMC4724860; doi:10.1177/0269881115622241)

## Supplementary Information

Results

Table S1

*Linear regression nested models for complete cases dataset displaying difference in IQ age 15 (SE) estimated between each cannabis user group compared to never users.*

| Cumulative cannabis use | IQ1 | IQ2 | IQ3 | IQ4 | IQ5a | IQ5b | IQ5c | IQ6 |
|---|---|---|---|---|---|---|---|---|
| <5 times | *-1.8 (1.0)** | *-2.1 (0.8)**** | *-1.7 (0.8)*** | *-1.7 (0.8)*** | *-0.5 (0.9)* | *-1.2 (0.8)* | *-1.6 (0.8)** | *-0.2 (0.9)* |
| 5-19 times | *-1.6 (1.3)* | *-2.7 (1.1)*** | *-2.8 (1.1)**** | *-2.9 (1.1)**** | *-1.0 (1.2)* | *-2.4 (1.1)*** | *-2.6 (1.1)*** | *-0.9 (1.2)* |
| 20-49 times | *-2.1 (1.8)* | *-3.3 (1.5)*** | *-3.1 (1.5)*** | *-3.3 (1.5)*** | *-1.0 (1.6)* | *-2.8 (1.5)** | *-2.8 (1.5)** | *-0.8 (1.7)* |
| ≥50 times | *-1.6 (1.8)* | *-2.9 (1.4)*** | *-2.5 (1.4)** | *-2.7 (1.4)** | *-0.2(1.7)* | *-1.4 (1.6)* | *-2.1 (1.6)* | *-0.1 (1.8)* |

* two-tailed t-tests, compared to never-users (p<.1)
** two-tailed t-tests, compared to never-users (p<.05)
*** two-tailed t-tests, compared to never-users (p<.01)

Model IQ1: Adjusted only by cumulative cannabis use at age 15
Model IQ2: As model 1 plus adjustment for full-scale IQ age 8
Model IQ3: As model 2 plus adjustment for maternal, early-life, and behavioural factors
Model IQ4: As model 3 plus adjustment for depressive symptoms and psychotic-like experiences
Model IQ5a: As model 4 plus adjustment for cumulative cigarette use at age 15
Model IQ5b: As model 4 plus adjustment for cumulative alcohol use at age 15
Model IQ5c: As model 4 plus adjustment for other recreational drug use at age 15
Model IQ6: As model 4 plus adjustment for cumulative cigarette use, cumulative alcohol use, and other recreational drug use at age 15

Table S2

*Linear regression nested models for complete cases dataset displaying difference in educational performance age 16 (SE) estimated between each cannabis user group compared to never users.*

| **Cumulative cannabis use** | **Ed1** | **Ed2** | **Ed3** | **Ed4** | **Ed5a** | **Ed5b** | **Ed5c** | **Ed6** |
|---|---|---|---|---|---|---|---|---|
| <5 times | *-2.9 (0.9)**** | *-3.1 (0.6)**** | *-2.7 (0.6)**** | *-2.7 (0.6)**** | *-0.7 (0.6)* | *-2.0 (0.6)**** | *-2.4 (0.6)**** | *-0.4 (0.6)* |
| 5-19 times | *-4.3 (1.2)**** | *-4.4 (0.8)**** | *-4.0 (0.8)**** | *-4.0 (0.8)**** | *-0.7 (0.9)* | *-3.1 (0.8)**** | *-3.1 (0.8)**** | *-0.2 (0.9)* |
| 20-49 times | *-8.0 (1.6)**** | *-5.6 (1.1)**** | *-4.3 (1.0)**** | *-4.3 (1.0)**** | *0.2 (1.1)* | *-3.2 (1.1)**** | *-2.6 (1.1)*** | *1.2 (1.2)* |
| ≥50 times | *-11.6 (1.5)**** | *-11.0 (1.1)**** | *-9.4 (1.0)**** | *-9.3 (1.0)**** | *-3.4 (1.2)**** | *-7.9 (1.1)**** | *-6.9 (1.1)**** | *-2.2 (1.3)** |

* two-tailed t-tests, compared to never-users (p<.1)
** two-tailed t-tests, compared to never-users (p<.05)
*** two-tailed t-tests, compared to never-users (p<.01)

Model Ed1: Adjusted only by cumulative cannabis use at age 15
Model Ed2: As model 1 plus adjustment for educational performance age 11
Model Ed3: As model 2 plus adjustment for maternal, early-life, and behavioural factors
Model Ed4: As model 3 plus adjustment for depressive symptoms and psychotic-like experiences
Model Ed5a: As model 4 plus adjustment for cumulative cigarette use at age 15
Model Ed5b: As model 4 plus adjustment for cumulative alcohol use at age 15
Model Ed5c: As model 4 plus adjustment for other recreational drug use at age 15
Model Ed6: As model 4 plus adjustment for cumulative cigarette use, cumulative alcohol use, and other recreational drug use at age 15

Table S3

*Fully adjusted model for complete cases dataset displaying difference in IQ age 15 estimated between each cigarette user group compared to never users.*

| Cumulative cigarette use | Adj. coef. | SE | t | p | 95% Cis | |
|---|---|---|---|---|---|---|
| | | | | | **Lower** | **Upper** |
| 1-4 times | *-0.85* | *1.06* | *-0.80* | *0.43* | *-2.93* | *1.23* |
| 5-20 times | *-1.81* | *1.10* | *-1.64* | *0.10* | *-3.97* | *0.36* |
| 21-60 times | *-2.55* | *1.23* | *-2.07* | *0.04* | *-4.97* | *-0.13* |
| 61-100 times | *-3.79* | *1.75* | *-2.16* | *0.03* | *-7.23* | *-0.35* |
| >100 times | *-3.22* | *1.36* | *-2.36* | *0.02* | *-5.89* | *-0.55* |

Table S4

*Fully adjusted model for complete cases dataset displaying difference in educational performance age 16 estimated between each cigarette user group compared to never users.*

| | | | | | 95% Cis | |
|---|---|---|---|---|---|---|
| **Cumulative cigarette use** | **Adj. coef.** | **SE** | **t** | **p** | **Lower** | **Upper** |
| 1-4 times | *-0.69* | *0.75* | *-0.93* | *0.35* | *-2.16* | *0.77* |
| 5-20 times | *-1.39* | *0.78* | *-1.80* | *0.07* | *-2.92* | *0.13* |
| 21-60 times | *-3.29* | *0.87* | *-3.79* | *0.00* | *-4.99* | *-1.59* |
| 61-100 times | *-5.32* | *1.23* | *-4.32* | *0.00* | *-7.73* | *-2.90* |
| >100 times | *-7.35* | *0.95* | *-7.72* | *0.00* | *-9.22* | *-5.49* |

Table S5

*Analyses comparing participants who were included in the complete case analysis to those with missing data. Total number of cases with missing data varies by variable due to varying degrees of missingness.*

| | Complete case | | Cases with missing data | | |
|---|---|---|---|---|---|
| | **N** | **%** | **N** | **%** | **p-value** |
| Female | *1194* | *53.4* | *5955* | *47.8* | *<0.001* |
| Mother has higher education | *1113* | *49.8* | *3279* | *32.2* | *<0.001* |
| Mother used cannabis in first 3 months of pregnancy | *37* | *1.7* | *280* | *2.7* | *0.003* |
| Mother in trouble with the law in 8 months following birth | *4* | *0.2* | *48* | *0.5* | *0.029* |
| Moved house after the child was born | *215* | *9.8* | *1326* | *15.2* | *<0.001* |
| Teacher reports child has played truant at age 10-11 years-old | *0* | *0.0* | *124* | *2.0* | *<0.001* |
| | **Mean** | **SE** | **Mean** | **SE** | **p-value** |
| IQ age 8 | *100.0* | *0.3* | *93.3* | *0.2* | *<0.001* |
| Maternal depression during pregnancy and up to 8 months old | *3.6* | *0.0* | *4.1* | *0.0* | *<0.001* |

Table S6

*Linear regression nested models for imputed dataset displaying difference in IQ age 15 (SE) estimated between each cannabis user group compared to never users.*

| | IQ1i | IQ2i | IQ3i | IQ4i | IQ5ai | IQ5bi | IQ5ci | IQ6i |
|---|---|---|---|---|---|---|---|---|
| <5 times | -2.1 (0.7)*** | -1.6 (0.5)*** | -1.3 (0.5)*** | -1.4 (0.5)*** | -0.4 (0.5) | -0.9 (0.5)* | -1.3 (0.5)** | -0.2 (0.5) |
| 5-19 times | -1.8(0.8)** | -2.0 (0.7)*** | -1.5 (0.6)*** | -1.6 (0.6)*** | -0.0 (0.6) | -0.9 (0.6) | -1.4 (0.6)** | 0.2 (0.6) |
| 20-49 times | -2.7 (1.3)** | -2.5 (0.9)*** | -1.8 (0.9)** | -1.9 (0.9)** | -0.1 (0.9) | -1.1 (1.0) | -1.6 (0.9)* | 0.3 (0.9) |
| ≥50 times | -3.1 (1.2)** | -2.8 (0.9)*** | -1.9 (0.9)** | -2.0 (0.9)** | -0.4 (1.0) | -1.2 (0.9) | -1.5 (0.9) | 0.6 (0.9) |

* two-tailed t-tests, compared to never-users (p<.1)
** two-tailed t-tests, compared to never-users (p<.05)
*** two-tailed t-tests, compared to never-users (p<.01)

Model IQ1i: Adjusted only by cumulative cannabis use at age 15
Model IQ2i: As model 1i plus adjustment for full-scale IQ age 8
Model IQ3i: As model 2i plus adjustment for maternal, early-life, and behavioural factors
Model IQ4i: As model 3i plus adjustment for depressive symptoms and psychotic-like experiences
Model IQ5ai: As model 4i plus adjustment for cumulative cigarette use at age 15
Model IQ5bi: As model 4i plus adjustment for cumulative alcohol use at age 15
Model IQ5ci: As model 4i plus adjustment for other recreational drug use at age 15
Model IQ6i: As model 4i plus adjustment for cumulative cigarette use, cumulative alcohol use, and other recreational drug use at age 15

Table S7

*Linear regression nested models for imputed dataset displaying difference in educational performance age 16 (SE) estimated between each cannabis user group compared to never users.*

| | Ed1i | Ed2i | Ed3i | Ed4i | Ed5ai | Ed5bi | Ed5ci | Ed6i |
|---|---|---|---|---|---|---|---|---|
| <5 times | *-3.7 (0.9)**** | *-2.2 (0.5)**** | *-1.4 (0.5)**** | *-1.4 (0.5)**** | *-0.1 (0.5)* | *-1.3 (0.5)*** | *-1.2 (0.5)*** | *-0.2 (0.5)* |
| 5-19 times | *-5.4 (1.2)**** | *-4.3 (0.9)**** | *-3.0 (0.9)**** | *-3.0 (0.9)**** | *-0.5 (1.0)* | *-2.8 (0.9)**** | *-2.6 (0.9)**** | *-0.6 (1.0)* |
| 20-49 times | *-7.3 (1.5)**** | *-4.7 (1.0)**** | *-2.8 (0.9)**** | *-2.8 (0.9)**** | *0.4 (1.0)* | *-2.5 (1.0)*** | *-2.1 (0.9)*** | *0.2 (1.0)* |
| ≥50 times | *-10.9 (1.4)**** | *-8.3 (0.8)**** | *-5.1 (0.8)**** | *-5.1 (0.8)**** | *-1.1 (1.0)* | *-4.7 (1.0)**** | *-4.2 (0.9)**** | *-1.3 (1.1)* |

* two-tailed t-tests, compared to never-users ($p<.1$)
** two-tailed t-tests, compared to never-users ($p<.05$)
*** two-tailed t-tests, compared to never-users ($p<.01$)

Model Ed1i: Adjusted only by cumulative cannabis use at age 15
Model Ed2i: As model 1i plus adjustment for educational performance age 11
Model Ed3i: As model 2i plus adjustment for maternal, early-life, and behavioural factors
Model Ed4i: As model 3i plus adjustment for depressive symptoms and psychotic-like experiences
Model Ed5ai: As model 4i plus adjustment for cumulative cigarette use at age 15
Model Ed5bi: As model 4i plus adjustment for cumulative alcohol use at age 15
Model Ed5ci: As model 4i plus adjustment for other recreational drug use at age 15
Model Ed6i: As model 4i plus adjustment for cumulative cigarette use, cumulative alcohol use, and other recreational drug use at age 15

Figure S1.

*Study participant flow diagram*

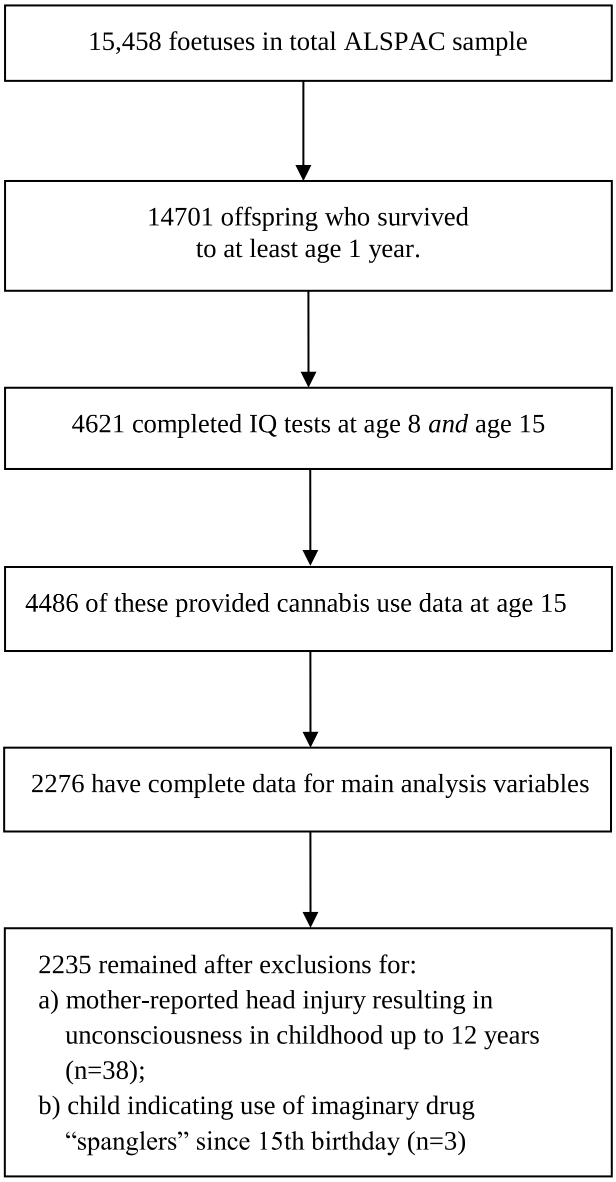

Supplement: Supplementary material [file JOP622241_-_Supplementary_material.pdf]
